# Some General Effects of War Upon the Voluntary Hospitals

**Published:** 1915-10-23

**Authors:** 


### SOME GENERAL EFFECTS OF WAR.*

### UPON THE VOLUNTARY HOSPITALS.

We have, in order to survey the general effect of the war upon voluntary hospitals, gathered the following facts from the published reports of nineteen typical institutions. This information, it will be observed, refers in most instances to the last five months of the year 1914.

### METROPOLITAN GENERAL HOSPITALS.

The London.—Occupied beds, 800. Although 250 beds were offered for soldiers, and 1,481 cases were treated up to December 31, there has by re-arrangement been but a small diminution in the number of civilian beds ordinarily and constantly used. Extra beds have been put up and the work of certain wards concentrated to prevent empties. The cost per occupied bed was increased. Many of the medical staff (honorary and paid) are serving; the hospital has reduced its research work in order to release doctors, 300 students and old Londoners are serving in various capacities with the Forces, as are 103 nurses and forty-five members of the lay staff, and the institution is using its organisation to train Army orderlies.

Structural works put off until more favourable times include the residents' hostel, aural wards, and the tuberculosis department. Work in connection with the laundry was proceeded with, as well as with the salvarsan wards (paid for by the Grocers' Company).

King's College.—Occupied beds, 332. Part of the hospital is now the 4th London General Military Hospital, and under military administration; thus the unopened wards in the new building are put to good use. The civilian portion continues under the ordinary management, and the Hospital Committee provides at scale rates everything—service and supplies—for the use of the soldiers. The main out-patient department is closed, but the casualty department is in full work, and also deals with some out-patients. The larger number of beds used had the effect of reducing the weekly cost per bed from £2 13s. 6d. to £1 18s. 9d.

Guy's.—Occupied beds, 522. One ward was used by Territorials and others who required some slight surgical treatment to enable them to enlist. 198 passed through in six months. The hospital offered to erect temporary buildings to accommodate 150 soldiers. Many medical, lay and nursing employees are serving. There was a small decrease in subscriptions, and an increase of £2,663 in ordinary expenditure (£2,000 approximately on provisions).

Great Northern Central.—Occupied beds, 168. The Convalescent Home at Clacton-on-Sea is used entirely for soldiers. 100 beds, some of them emergency beds, were provided at the hospital, and 159 cases passed through up to the end of the year. "Considerable difficulty in obtaining medical residents," and "a considerable number of nurses" are serving. Ordinary expenditure apparently unaffected.

St. George's.—Occupied beds, 314. One hundred beds at hospital and convalescent home set aside for wounded; 105 cases passed through. Nearly all resident medical staff left to serve, and also a considerable number of nurses. Hospital much helped by old and experienced house officers. Decrease in subscriptions, £250.

St. Thomas's.—Occupied beds, 351. Two hundred extra beds placed in civilian wards and inconvenience reduced to a minimum; 124 passed through. Ward for 12 officers, 86 treated. One hundred and five Canadian nurses housed for five weeks. Temporary wards now erected in grounds. Many members of honorary medical staff attached to No. 2 Territorial Hospital, others with Forces. Plans for new out-patient department held up. Increase in cost of provisions, £1,705; surgery and dispensary, £1,183. Increase in cost of meat alone, £750.

St. Mary's.—Occupied beds, 249. Special feature: manufacture and supply of vaccines to Allied Armies. Up to date of issue of report 4,580,000 doses of anti-typhoid, cholera, and other vaccines prepared and supplied. One hundred beds offered "in the event of accommodation in military hospitals proving inadequate." Soldiers began to arrive in November. Majority of honorary medical staff called up, three abroad. Depletion of resident medical and nursing staffs; eight of lay staff serving. No extraordinary expenditure incurred.

Middlesex.—Occupied beds, 306. Two hundred and ten beds offered to War Office (80 in hospital and 130 at Clacton-on-Sea branch, where accommodation was increased from 95 to 130). The Chairman, Prince Alexander of Teck, is serving at the Front. Small loss in subscriptions. Increase under all headings of expenditure.

Metropolitan.—Occupied beds, 111. Twenty-four beds offered and fully used. Shortage in resident medical staff. Several members of honorary staff serving, also a number of nurses. Small reduction in cost per occupied bed.

St. Bartholomew's.—Occupied beds, 556. One hundred and seventy four beds offered and 1,039 (up to date of report) passed through. Twenty-seven honorary medical staff in Territorial hospitals, others serving with Forces. Resident medical staff at once depleted. One hundred present and nearly 800 past students serving, also 77 nurses and 34 lay employees. To meet medical service difficulties O.P. special departments have reduced number of days, and board (costing £20 a week) is now provided for some resident staff formerly not allowed board. £466 less in subscriptions and donations, £925 increase in provisions for five months.

* N.B.—Evidence of the effects of war upon other sections of the field of charity is invited.—Ed. "The Hospital."
The previous article appeared on October 16.

METROPOLITAN SPECIAL HOSPITALS.

Mount Vernon.—Occupied beds, 104. Building of Nurses' Home postponed. £260 reduction in annual subscriptions. Increase in expenditure under many headings.

National Hospital for the Paralysed and Epileptic.—Occupied beds, 143. Provision for "a certain number" of soldiers with nerve injuries and nervous complaints. Several honorary and resident medical staff and nurses serving. Small increase in expenditure. Decrease in annual subscriptions.

Royal Hospital for Diseases of the Chest.—Occupied beds, 66. Some honorary staff serving. Small increase in expenditure. Falling off of £400 in annual subscriptions in four years.

Royal London Ophthalmic.—111 occupied beds. Thirty beds offered to War Office. Large number of soldier out-patients treated. Several hon. medical staff, nurses, and lay staff serving. For four years in succession income has fallen short of expenditure. Decrease of £22 in subscriptions.

PROVINCIAL HOSPITALS.

Royal Victoria Infirmary, Newcastle-upon-Tyne.—Occupied beds for civilians, 378 (411 in 1913); 200 beds for wounded. Out-patient hall used as ward to take 114, and two special pavilions. Thirteen resident medical staff and fifteen nurses serving. 8,000 fewer out-patients. Boarded 90 nurses of 1st Northern General Military Hospital. Workmen's contributions increased (now £20,756). Cost per occupied bed, up £4 per annum. Consideration of building Nurses' Home, massage department, and balconies postponed.

Radcliffe Infirmary, Oxford.—148 occupied beds. One ward taken and temporary building used for wounded, 60 beds in all. Depletion in resident medical staff. Ten nurses serving. Slight increase in cost of provisions. Increase in subscriptions.

Devonshire Hospital, Buxton.—270 occupied beds; 150 beds offered for soldiers suffering from rheumatism and allied complaints: 279 admitted.

Glasgow Royal Infirmary.—Occupied beds, 678. 150 beds offered for wounded, 327 cases received. Twenty-one medical and surgical staff and many nurses serving. Ordinary expenditure increased from £51,864 to £56,026. Subscriptions increased £337. Rebuilding of medical school postponed.

Liverpool Royal Infirmary.—Occupied beds, 265. Fifty beds provided for wounded. Nearly whole of honorary medical staff attached to General Military Hospital at Fazakerley. Difficulty in maintaining numbers of resident medical staff. Sixteen nurses serving. Increases in numbers of in-patients and out-patients. Number of suitable candidates as nursing probationers "largely increased." Expenditure and income have both fallen slightly. Subscriptions, small increase. Rebuilding of Nurses' Home postponed.

In most of the hospitals under review facilities for training V.A.D. St. John Ambulance workers have been provided.

Income and Expenditure.

It will be gathered from the instances taken that the effect on the income and expenditure of hospitals had not yet been fully felt up to the close of last year. This is probably so because the greater part of the income is generally received during the first half of the year, and existing contracts (even when modified) would cover the main supplies at reasonable prices until after the period reviewed. The effect on expenditure in 1915 will clearly be very different, judging the situation in the light of present prices and conditions of service.

From the reports quoted above, one of the facts especially notable is that everything that can be avoided or postponed in the shape of extraordinary expenditure has, very sensibly, been placed on one side until more settled times.

Every student of institutional statistics must look forward with interest to tabulated information in respect of the returns for the current year.

